# Improving the Robustness of Complex Networks with Preserving Community Structure

**DOI:** 10.1371/journal.pone.0116551

**Published:** 2015-02-12

**Authors:** Yang Yang, Zhoujun Li, Yan Chen, Xiaoming Zhang, Senzhang Wang

**Affiliations:** School of Computer Science and Engineering, Beihang University, Beijing, China; University of Maribor, SLOVENIA

## Abstract

Complex networks are everywhere, such as the power grid network, the airline network, the protein-protein interaction network, and the road network. The networks are ‘robust yet fragile’, which means that the networks are robust against random failures but fragile under malicious attacks. The cascading failures, system-wide disasters and intentional attacks on these networks are deserving of in-depth study. Researchers have proposed many solutions to improve the robustness of these networks. However whilst many solutions preserve the degree distribution of the networks, little attention is paid to the community structure of these networks. We argue that the community structure of a network is a defining characteristic of a network which identifies its functionality and thus should be preserved. In this paper, we discuss the relationship between robustness and the community structure. Then we propose a 3-step strategy to improve the robustness of a network, while retaining its community structure, and also its degree distribution. With extensive experimentation on representative real-world networks, we demonstrate that our method is effective and can greatly improve the robustness of networks, while preserving community structure and degree distribution. Finally, we give a description of a robust network, which is useful not only for improving robustness, but also for designing robust networks and integrating networks.

## Introduction

Many complex systems in nature can be represented as networks [1–5]. These include essential infrastructures and systems, including power grid, airline networks and protein interaction networks. Research have however shown that many of these networks are ‘robust yet fragile’ [6–13]. This seems like an oxymoron, but what it really means is that some vertices, which are called “hubs", have many more connections than others. Hence, the malicious threats and attacks on the hubs make the network fragile, however the failures and attacks on the randomly chosen vertices make the network robust. In fact, the attacks or errors on networks are not limited to the deletion of vertices. Four common measures can make the network more fragile: vertex deletion [14, 15], edge deletion [16, 17], vertex addition and edge addition [18]. The addition of vertices or edges on networks may make some important edges or vertices more important. This will increase the risk of the attacks on these important vertices or edges. The deletion of edges [17, 19, 20] is also a common attack measure, however the deletion of vertices with their linked edges is more harmful. Hence, we focus on the deletion of vertices on networks in this paper. To mitigate the attacks on vertices, researchers have proposed many solutions [21–23] to improve the robustness of complex networks. These solutions typically involve a re-configuration of the network edges. Besides robustness, such re-configurations affect two other key characteristics of these networks, namely the degree distribution and community structure of the networks. The degree distribution clearly captures a amount of information about a network which gives important clues into structure of a network. The community structure [16, 24, 25] refers to the functional modules in networks which can provide insight into how network function and topology affect each other. When a network is re-configured to improve its robustness, we should still aim to retain as much of the original defining characteristics of the original network as possible. This is because these characteristics define the very nature and functionality of the network, and altering these drastically may cause the network to lose its intended purpose. Take for example a power grid. In the United States, population growth and settlement is not consistent through the whole country. Much of the population can be found concentrated in major cities along the east and west coasts. As such, power grids are also configured such that more power is delivered to the coasts. If we want to improve the robustness of the network, we could perhaps reassign links such that power distribution is more even throughout the entire country, but this is not practical because more power is needed where more people are housed nearby. Solutions that re-configure the power grid network for better robustness thus cannot ignore its inherent degree distribution and community structure. The main approaches to improve network robustness can be generally classified into three main categories.

The first category involves the addition of edges to existing networks [26, 27]. While having additional edges introduces redundancies and improves network robustness, in real life it may not be very practical. This is because the new edges change the degree distribution of a network. Moreover, adding many edges to a network may dramatically change the community structure of the network.

Instead of adding new edges, the second category of solutions seeks to make networks homogeneous (i.e., ensure that all vertices have similar degrees) by reconnecting edges. Hema et.al. [28] proposed a random neighbor rewiring scheme which disconnects an edge connected to a high degree neighbor and reconnects it to another random node, which would be a lower degree node given the power law nature of the scale-free graphs. Effective as this may be, it requires that most of the connections in a network be reset. Also, it also affects the degree distribution and community structure of the network.

**Fig 1:**
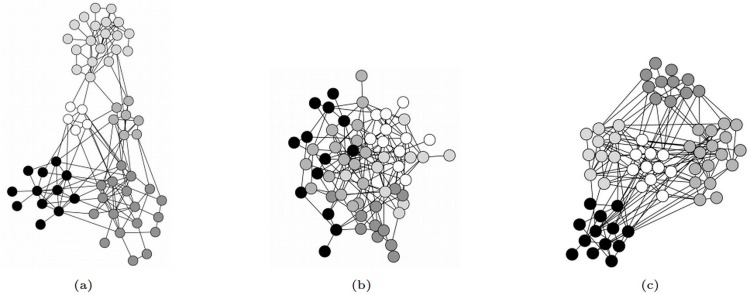
The change of community structure after applying the schneider’s method [23] on the dolphin network. Fig. 1(A) presents the community structure of the dolphin network with N = 62 dolphins and M = 159 co-appearance of them. The network can be divided into 5 communities, which are marked as different colors. Fig. 1(B) presents the onion-like structure of the improved network. The community structure of the network is greatly changed. Fig. 1(C) shows the clear community structure of Fig. 1(B), i.e. the improved network. The first two figures are drawn by the Gephi automatically, while the vertices in the last figure are separated by the colors manually. The social network of 62 bottlenose dolphins was observed by Lusseau [30]. The dolphins lived in Doubtful Sound, New Zealand. Lusseau collected the data of dolphins according to his field studies of dolphins for two years. The ties between dolphin pairs are established by the observation of the statistically significant frequent association.

The third category for robustness improvement seeks to overcome the problems of the earlier methods by swapping two randomly chosen edges. For instance, the edges $e_{ij}$ and $e_{kl}$, which connect node i with j, and node k with l, respectively, become $e_{ik}$ and $e_{jl}$, only if the robustness of the network is increased; i.e., *R*_*new*_>*R*_*old*_ [23, 29]. With this solution, the robustness of a network can be significantly improved while maintaining its degree distribution. However this still affects the community structure of the network. Take the dolphin social network [30] as an example. After applying the third category method, the original network in Fig. 1(A) turned into the improved network which is an onion-like structure under HDA. As shown in Fig. 1(B), the structure consists of a core of highly connected vertices hierarchically surrounded by rings of vertices with decreasing degree. In the improved network, vertices with high degree connect with each other, and vertices with low degree connect with each other. Fig. 1(C) represents the clear community structure of Fig. 1(B). Though the number of the communities is unchanged, the community structure and the size of each community is greatly changed. The improved networks turn into an ‘onion-like’ topology, while the community structure of the original network is dramatically changed.

In our work, we seek to improve the robustness of networks whilst retaining as much of their defining characteristics as possible. To more effectively quantify our approach, we attribute a cost to the changes that can be applied onto a network, and aim to reduce these costs in our approach. These changes include:

- The addition of edges. For example, adding edges(Fiber optics) between nodes(continents) in the optical network will cost too much.
- The change of degree distribution. The change of degree distribution will rewire lots of connections of the network. In real world networks, the modification of connections on the power grid network and road network is a huge waste of money and time.
- The change of community structure. Community structure actually plays an important role in many aspects [16, 31–35], such as the cascaded failures [36] and disease dynamics [37]. Salathe et al. [37] have found that community structure can provide invaluable help in understanding the function of networks. The change of community structure leads to the loss of functional modules, especially in the protein-protein interaction network and disease-gene network [38–40]. Hence, how to prevent the function failure of networks in improving the robustness is an important issue to be resolved.

To improve robustness while minimizing the above three costly changes, we first seek to verify that the community structure of networks actually do identify the robustness and vulnerability of networks to some extent. Then, we propose an effective 3-step strategy for robustness improvement, which retains the degree distribution of a network, as well as preserves its community structure. Our proposed method can keep together the integrity of networks under both random and targeted attacks, even when the networks are inflicted with significant damages. Further, our proposed method can provide good suggestions on how we can generate robust networks with a given degree distribution and community structure.

## Materials and Methods

### Robustness Measure

Traditional robustness measures ignore situations in which the network suffers a big damage without completely collapsing. Therefore, schneider et al. [23] proposed a robustness measure *R*, which considers the size of the largest component during all possible malicious attacks for undirected networks:

$$R=\frac{1}{N}\sum_{Q=1}^{N}s^{\prime }\left(Q\right)$$ (1)

where *N* is the number of vertices of the network and $s^{\prime }$ *(Q)* is equal to *s(Q)/(N-1)*. *s(Q)* is the number of vertices in the largest connected component after removing *Q = qN* vertices, and is normalized by *N-1*, where *q* is the fraction of damaged vertices in the network. While *q* ranges from *1/N* to *N/N*, the corresponding *Q* ranges from 1 to *N*. The sum of $s^{\prime }$ *(Q)* is divided by the number of the vertices *N* for the purpose of normalization. The minimum of *R* is equal to *1/N*, when the network is a star network. The maximum of *R* is equal to 0.5, when the network is a complete network. This distinctive measure is not only simple but also practical, due to the calculation of the size of the largest component during all possible system-wide failures or intentional attacks.

### Attack Procedure

The attack procedure is to remove vertices according to the order of their importance. For instance, we can evaluate the importance of vertices respectively by utilizing the degree, betweenness, closeness, katz, or eigenvector centrality measure, and then compute the effect on the size of the largest connected component of the network after removing a given fraction of the vertices in decreasing rank order with respect to the specified centrality measures. There exists two distinct attack strategies. The first one is the simultaneous targeted attack. The centrality measure is calculated for all vertices in the network, and then a specified fraction of the vertices are removed in order of the centrality measure, from highest to lowest [22]. The other attack strategy is the sequential targeted attack. The centrality measure is calculated for all vertices in the original network, and the vertex with highest centrality value is removed. The removal of this vertex results in a new network in which the centrality ranking of the remaining vertices may be different from the previous ones. Thus, we recalculate the centrality measures of all vertices in the new network and remove the highest one again [22].

It is worth mentioning that degree centrality is a more effective means of targeting vertices than any of the other centrality measures under simultaneous targeted attack. The second attack strategy “sequential targeted attack" is a more harmful and natural strategy than the “simultaneous targeted attack”. Under sequential targeted attack, there are small differences in the attack effect for different centrality measures [22]. Consequently, sequential attack strategy is adopted in this paper, and the vertices are mainly removed in decreasing order of degree centrality, i.e., High Degree Attack(HDA). In the Supporting Information (S1 File), we take other centrality measures into consideration.

### Community Structure and Robustness

Before we introduce the 3-step strategy for robustness improvement while keeping the community structure of the network, it’s worthwhile to interpret the relationship of robustness and community structure. The quality of community structure is usually measured by the modularity index [16]. Hence, in this section, experiments are designed to illustrate the relationship of robustness and modularity.

**Fig 2:**
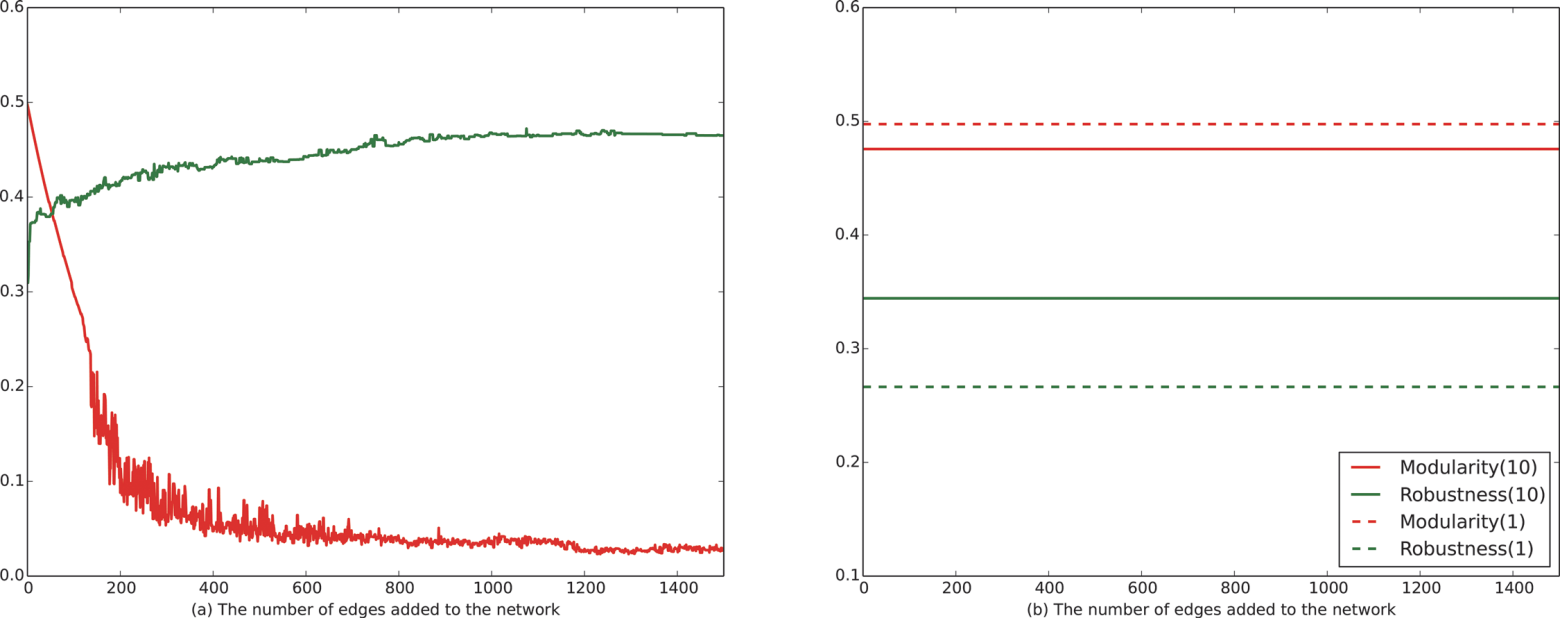
The relationship of community structure and robustness. In Fig. 2(A), 2000 edges are added to the network to connect two ER random networks. The robustness of the network is greatly improved, while the modularity of the network is a linear decreasing function of the number of added edges at the beginning. After only 4 edges are added to the network, the robustness of the network is greatly changed. After 200 edges are added to the network to connect two ER random networks, the modularity of the network decreases by 80%. In Fig. 2(B), two ER random networks are firstly connected by 10 edges, and then 2000 edges are added to the network to randomly connect the vertices in the same community. The robustness and modularity of the network is stable. When the two ER random networks are connected by only 1 edge, the phenomenon is the same. When two ER random networks are connected by 50 edges, the values of modularity and robustness are both approximately equal to 0.38. If two ER random networks are connected by more edges, the robustness continues to increase and the modularity continues to decrease. The increase of robustness is faster than the decrease of modularity.

Two ER random subnetworks [41] of locally dense communities with 50 vertices and 200 edges are created. On the one hand, we randomly connect the vertices in different communities by adding edges among the two ER subnetworks. As the number of edges that connect different communities increases, as shown in Fig. 2(A), the robustness of the network rises sharply at the beginning, and then rises slowly. After only 4 edges are added to the network, the robustness of the network rise conspicuously. In contrast to the robustness, the modularity of the network drops linearly at the beginning, and then drops slowly after 250 edges are added to the network. On the other hand, we randomly connect two different communities with 10 edges and then randomly connect the vertices in the same community with 2000 edges. Fig. 2(B) shows that the modularity and the robustness of the network change inconspicuously. Once the number of edges which connect with different communities is determined, the robustness of the network is determined, no matter how many edges are added into the community. The same phenomenon exists when we randomly connect two different communities with only 1 edge in Fig. 2(B).

In a word, real networks with weak community structure(small modularity) are more robust than that with strong community structure [16]. The above conclusion makes sense, in that networks with strong community structure have few edges which connect different communities. Hence, the network with strong community structure is fragile in consideration of the attack on those edges. In addition, if the cost of adding edges in the network is low, the most easy and effective way to improve the robustness of the network is to figure out the community structure and add few edges between different communities. In conclusion, the limitation of the robustness improvement depends on two aspects: the internal structure of the community and the number of edges that lie between communities. Based on this, we propose our method for robustness improvement in the next section.

### 3-step Strategy for Robustness Improvement

To improve the robustness of networks without greatly altering community structure and keeping the degree distribution of the networks, we propose the following method. The main idea is to make the internal structure of each community represent onion-like structure and connect different communities with low importance vertices. The importance of vertices is determined by the centrality measures. Under HDA strategy, the importance of vertices is calculated by degree centrality.

Step 1. Make each community represent onion-like structure, i.e., swap edges to make the vertices which have similar importance connect with each other.
Step 2. Swap edges to make the vertices with high importance only connect with the vertices in the same community.
Step 3. Properly swap edges to increase the number of edges among communities(optional step).

**Fig 3:**
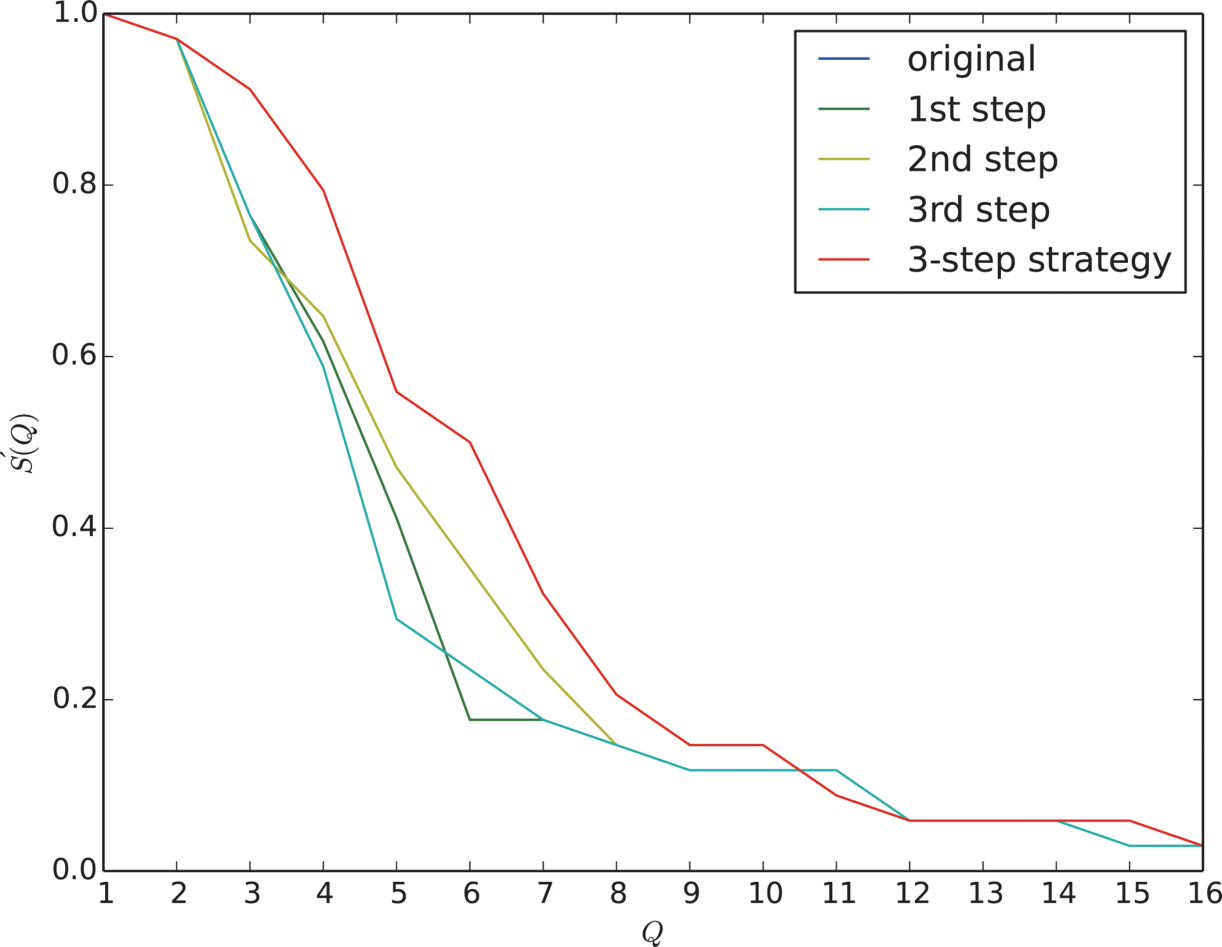
Four approaches are respectively applied on the karate network for robustness improvement. Our approach outperforms other single step methods in sector [1,10]. When the network suffers a severe damage, i.e., 85% of vertices are attacked, all the methods fail to improve the robustness. At the beginning, the 1st step and the 3rd step works well. After that, the 2nd step successfully mitigate the attacks. Our strategy combines the 3 steps together and obviously make the network more robust. The sharp drop of curves represents that the network breaks into several components and we will introduce the details in the rest of this section. The karate club analyzed by Zachary consists of 34 persons. If person A is a friend of person B, there is an edge between A and B. There are 78 edges in this club. The friendship relationships are investigated over two years. During two years of follow-up, Zachary et al. found that the club split into two clubs, due to a conflict between an administrator and a coach in the club.

The motivations and details of each step are introduced in Supporting Information (S1 File) text. The above 3 steps can be recursively used for robustness improvement until the robustness is hard to rise. The third step is an optional step. If the cost of this step is not high, we can adopt this step for many times. To briefly introduce the effectiveness of our method, we take a small real world network, i.e., the karate network [42] as an example. In Fig. 3, step 1, step 2 and step 3 are separately applied for robustness improvement. The step 1 and the step 2 can release the damage of the attack in the sector [2,4]. The effectiveness of the ‘1st step’ is slightly better than the ‘2nd step’ in this sector. The curve of ‘3rd step’ almost coincides with the curve of ‘original’. By analyzing the experimental results, we find that the curve of ‘3rd step’ is slightly higher than the curve of ‘original’. In sector [4,8], the second step performs well. All 3 steps are applied on the karate network in mitigating the malicious attack on the network. The red curve in Fig. 3 clearly shows that the robustness of the karate network is greatly improved. The robustness of the karate network increases from 0.1263 to 0.1773, increases by 40.4%.

**Fig 4:**
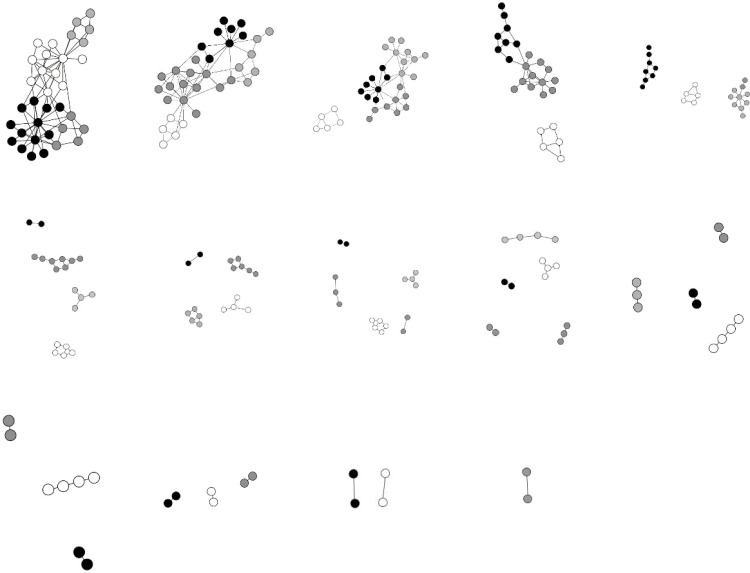
The process of the malicious attack on the karate network.

**Fig 5:**
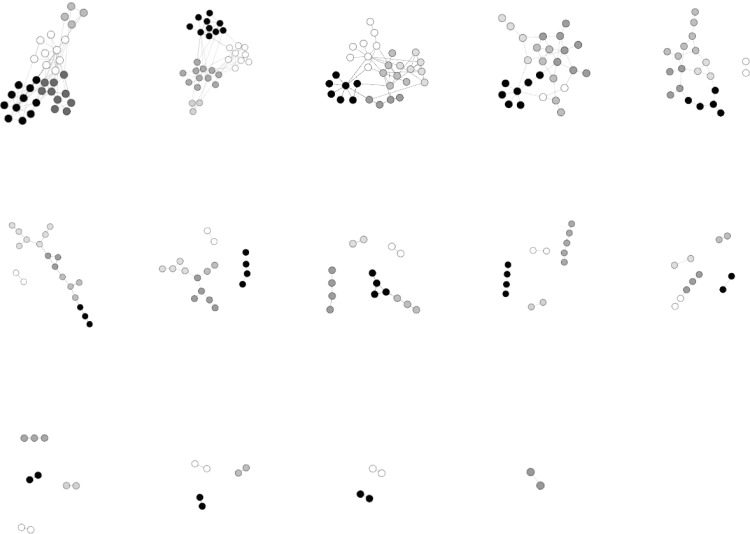
The process of the malicious attack on the improved karate network.

An interesting phenomenon is that each curve falls sharply for two stages. To find out what happened when the curves drop, we draw the attack process on the original network and the improved network in Fig. 4 and Fig. 5. The components which contain more than 2 vertices are drawn in the figure. To the original network, the curve firstly falls in sector [1,2] which corresponds to the sub-figure 1 to 2 in Fig. 4, and then falls sharply in sector [2,4] and sector [4,9]. In sector [2,3], the attack breaks the network into 2 components. In sector [4,9], the attack breaks the network into 5 components. To the improved network, the two sharp falls lie in sector [2,5] and [5,9]. After 3 deletions of vertices on the original network, the attack breaks the karate network into 2 components. By contrast, the improved karate network can withstand 5 attacks, and then breaks into 2 components. According to the figure, the fall of robustness is greatly mitigated, especially in sector [2,5] and [5,9].

## Results

### Improving Existing Infrastructures

**Fig 6:**
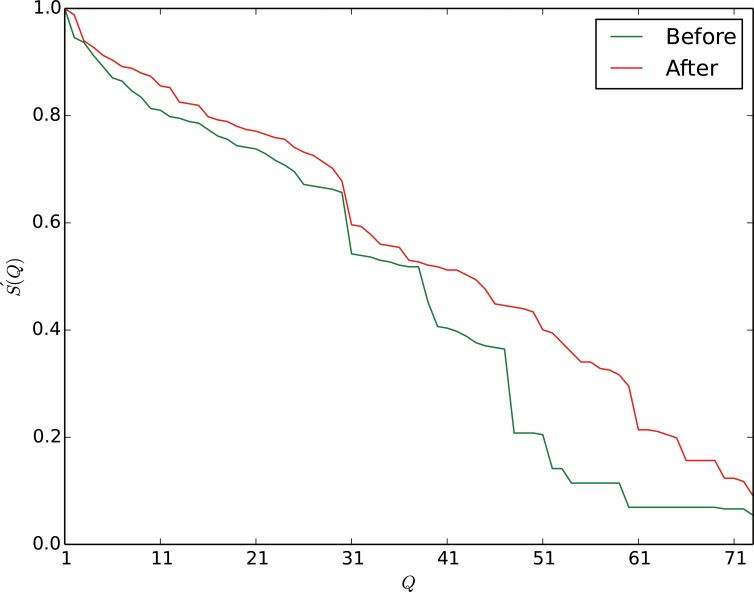
3-step strategy on the USA airport transportation network. There is no sharp drop in sector [1,31]. The two curves in this sector show that the 3-step strategy succeeds in improving the inner structure of community. In sector [31,51], we can see that the 3 sharp drops are greatly mitigated. It means that the proposed strategy successfully defers that the network breaks the network into components. The mitigation of malicious attack on the airline network is obvious, especially when the network suffers considerable damage. The US airline network consists of 332 vertices and 2126 edges in 1997, which has similar behavior to the BA [10] networks. If two US airports have a direct flight connection, there is an edge between them.

The Girvan and Newman’ method [16] is applied on the original network and the improved network to detect communities. Then, we evaluate our robustness improvement cost by using the concept of community unchanged ratio, which is defined as the number of vertices that their community unchanged divided by the total number of vertices. On the karate network, despite the strong constraints, the robustness R of the karate network can be increased by 40.4%, and the community unchanged ratio is 85%. On the USA airport transportation network, as shown in Fig. 6, the robustness R of the airline network can be increased by 48.6% with 91% vertices’ community unchanged. It indicates that our optimized network is not only more robust against malicious attacks, but also does not significantly change the modularity without any loss of functionality.

### Designing Robust Networks with Community Structure

According to the analysis in this paper, a description of robust network is provided in this section, which are useful guidelines in designing robust networks with community structure. The characteristic of a robust network with community structure is as follows. Each community in the network represents an onion-like structure, in which the vertices preferentially connect with the vertices with similar importance. As shown in Fig. 1(B), the most important vertices lie in the middle of the community surrounded by the less important vertices connect with each other. The connections among different communities is that the least important vertices of different communities connect with each other, i.e., the vertices with high importance only connect with the vertices in the same community.

If there exists numerous communities in the network, how to connect these communities is a meaningful task. After making each community represent an onion-like structure, we can treat each community as vertices and reapply our algorithm. Hence, the structure of numerous communities is also an onion-like structure. In addition, the description above can provide good suggestions in integrating two networks, e.g. the sensor networks, the power grid networks, which can guarantee the robustness of the integrated network.

### Structural Diversity in the Original and Improved Network

**Fig 7:**
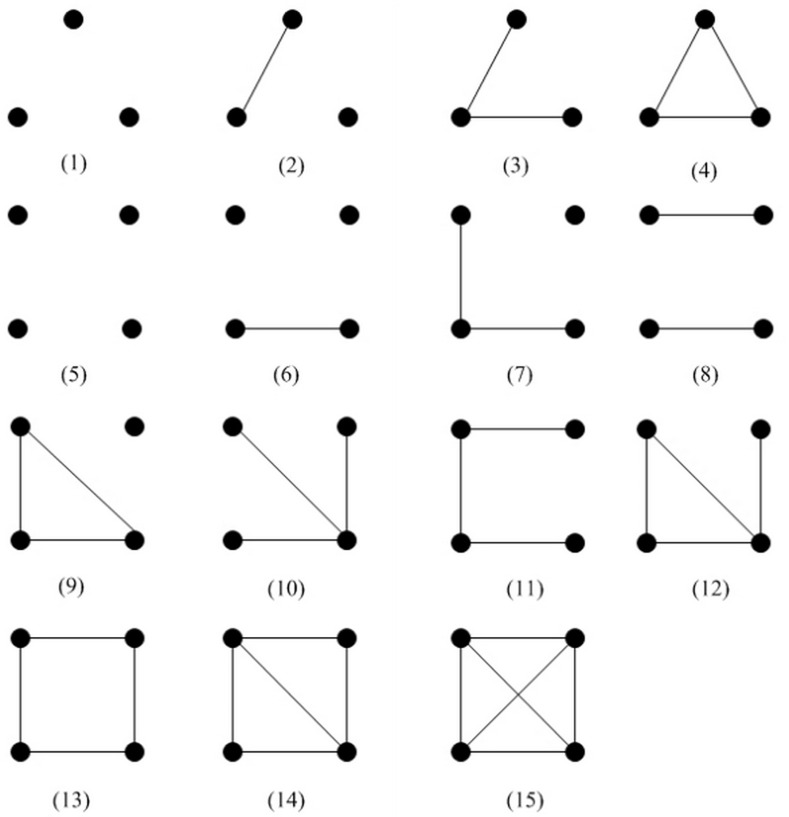
All 3-node and 4-node subgraphs.

**Fig 8:**
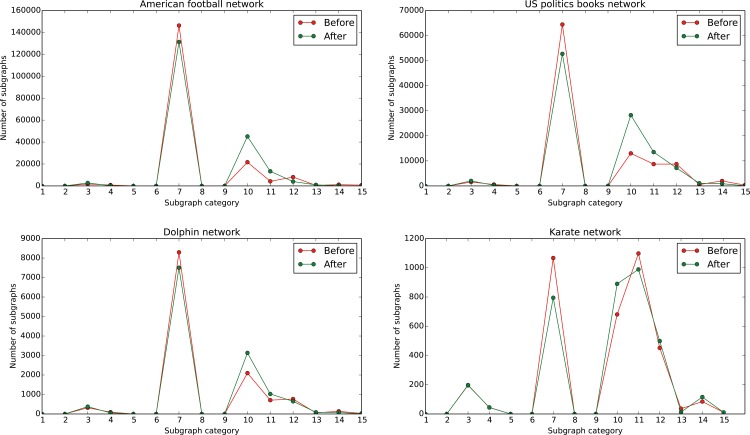
Structural diversity in the football, US politic books, dolphin and karate network and the corresponding improved network. This figure indicates that real networks mainly consists of only 4 subgraphs-7,10,11,12.

In this part, the relationship of the robustness and structural diversity is interpreted. Fig. 7 illustrates the 4 types of 3-node subgraphs and 11 types of 4-node subgraphs. Structural diversity [43, 44] is the distribution of all possible 3-node and 4-node subgraphs, as shown in Fig. 8. According to Jon Kleinberg [43] and Yang’s [45] opinion, the networks are constructed by 3-node and 4-node subgraphs. Consequently, the distribution of these subgraphs and the connections of them play an important role in identifying the robustness of the network.

The numbers under the horizontal axis in Fig. 8 correspond to the subgraphs in Fig. 7. According to the figures, the most frequently observed subgraphs in the networks are subgraph 7,10,11,12. The difference of structural diversity between the original network and the improved network is presented in Fig. 8, which indicates that robust network have less subgraph 7 and more subgraph 10 and 11. This phenomenon makes sense in that more subgraph 10 and 11 increase the correlation of edges in the graph. The phenomenon also exists when we apply schneider’s method on the network. According to the analysis above, adjusting the distribution of 3-node and 4-node subgraphs to increase the edge correlation is an effective approach in designing robust networks.

## Discussion

In conclusion, we firstly interpret the relationship of community structure and robustness in this paper, then propose a novel scheme that can significantly improve the robustness of network, while keeping its degree distribution and community structure under several attack strategies. The experimental results show that the new scheme is successfully useful on many real world networks. Secondly, the structural diversity is introduced, which indicates that micro-structures play an important role in identifying the robustness of networks. Lastly, we give a detailed description of the structure of robust network. In this case, our measure can provide good guidelines not only in improving the existing networks but also in designing the robust network with community structure. In addition, the conclusion of our method is helpful in integrating two networks. In the future, our work will focus on analyzing how the connections of different subgraphs determine the robustness of the network. In addition, according to the experimental results, we can infer that the most harmful way to attack networks with strong community structure is firstly attacking the structure hole spanners [46] but not the most important vertices determined by centrality measures. For the networks with very weak community structure, the most harmful strategy to attack them is using the betweenness and degree centrality measures. Furthermore, whilst present works focus on the attacks on the vertices, the attack on the connections or edges is a meaningful issue to be resolved.

## Supporting Information

S1 File Combined Supporting Information File. Text A: Detailed Description of the 3-step Strategy. Text B: Other Attacks. Text C: Properties of the Original and Improved Networks. Text D: Small Density vs Large Density. Figure A: Robustness against betweenness, closeness and HITS attack on the dolphin network and the improved network. Betweenness is the most harmful attack strategy. After only 5 attacks, the network breaks into pieces. In contrast to the betweenness attack strategy, the network can withstand 7 closeness attacks before it breaks into pieces. Closeness is a more harmful attack strategy than HITS. The reason is that HITS compiles two ranking vectors: hubs and authorities, however, on undirected networks, the hubs and authorities gain the same score, while the scores for hubs and authorities are different when the network is directed. Table A: Properties of Original and Improved Networks.
(PDF)
