# Supplementary material for: Improving the Robustness of Complex Networks with Preserving Community Structure
Source: PLoS One. 2015 Feb 12;10(2):e0116551. doi: 10.1371/journal.pone.0116551 (PMC4326464; doi:10.1371/journal.pone.0116551)
Supplement: S1 File — Text A: Detailed Description of the 3-step Strategy. Text B: Other Attacks. Text C: Properties of the Original and Improved Networks. Text D: Small Density vs Large Density. Figure A: Robustness against betweenness, closeness and HITS attack on the dolphin network and the improved network. Betweenness is the most harmful attack strategy. After only 5 attacks, the network breaks into pieces. In contrast to the betweenness attack strategy, the network can withstand 7 closeness attacks before it breaks into pieces. Closeness is a more harmful attack strategy than HITS. The reason is that HITS compiles two ranking vectors: hubs and authorities, however, on undirected networks, the hubs and authorities gain the same score, while the scores for hubs and authorities are different when the network is directed. Table A: Properties of Original and Improved Networks. (PDF) [file pone.0116551.s001.pdf]

File S1. Supporting Information

Yang Yang[1], Zhoujun Li[1,*], Yan Chen[1], Xiaoming Zhang[1], Senzhang Wang[1]
1 School of Computer Science and Engineering, Beihang University, Beijing, China
∗ **E-mail: lizj@buaa.edu.cn**

CONTENTS

Text A. Detailed Description of the 3-step Strategy
Text B. Other Attacks
Text C. Properties of the Original and Improved Networks
Text D. Small Density vs Large Density
Figure A. Robustness against betweenness, closeness and HITS attack on the dolphin network and the improved network. Betweenness is the most harmful attack strategy. After only 5 attacks, the network breaks into pieces. In contrast to the betweenness attack strategy, the network can withstand 7 closeness attacks before it breaks into pieces. Closeness is a more harmful attack strategy than HITS. The reason is that HITS compiles two ranking vectors: hubs and authorities, however, on undirected networks, the hubs and authorities gain the same score, while the scores for hubs and authorities are different when the network is directed.
Table A. Properties of Original and Improved Networks
References A.

Text A. Detailed Description of the 3-step Strategy

The detail of the 3-step strategy is as follows. The purpose of the first step is to make each community represent a onion-like structure, which is a significant character of robust structure [1, 2]. Hence, we rank the nodes according to their importance first, and then swap edges to make the vertices which have similar importance connect with each other. Four nodes i,j,k,l are randomly chosen in the same community. e_{ij} and e_{kl} are the edges among the four nodes. If the swap of the edges neither creates self-connections nor double-connections, this swap is accepted. The purpose of the second step is to avoid the "bridges", which connect different communities, being attacked firstly. If the "bridges" disappear, the robustness of networks declines sharply. Moreover, important vertices are always attacked firstly. Hence, we swap edges to make the vertices with high importance only connect with the vertices in the same community. The purpose of the third step is to properly add connections among different communities. The swap of edges in this step should obey the principal of the first two steps. Each step is recursively done for many times until the robustness of the network increases inconspicuously.

Text B. Other Attacks

On the basis of the 3-step strategy, under sequential targeted attack, our robustness optimization scheme works well against other attack strategies. Fig.A presents the betweenness, closeness and HITS (Hyperlink-Induced Topic Search) centrality attack strategy in destroying the dolphin network. The robustness of the network is greatly improved. Betweenness centrality is the most effective way to attack networks, while HITS centrality is an ineffective way to attack the network. Consequently, the improvement of our scheme on the network is inconspicuous. Under the betweenness attack, the 3-step strategy improves the robustness of the dolphin network by 31.6% with community unchanged ratio 93%, while under closeness attack the 3-step strategy increases the robustness of this network by 22.4% with community unchanged ratio 89%. Under the HITS attack, the 3-step strategy improves the robustness by 7.5% with community unchanged ratio 92%.

Text C. Properties of the Original and Improved Networks

In Table A, we show the clustering coefficient, network diameter, network centralization and characteristic path length, robustness and modularity of the original and improved networks. As the robustness increases, both characteristic path length and clustering coefficient decrease, while other properties of the networks don't change significantly.

Text D. Small Density vs Large Density

We have designed and implemented the two experiments to find whether the proposed method works well if we change the connection density of different communities.

- Experiment 1 – small connection density case:

We generate two ER networks with 50 vertices and 100 edges. Two ER networks are randomly connected by 10 edges. Then after applying the 3-step strategy to improve the robustness, the robustness of network increases from 0.2209 to 0.2798 (increases by 26.7%), and the modularity of the network decreases from 0.5054 to 0.4296.

- Experiment 2 – large connection density case:

We generate two ER networks with 50 vertices and 1000 edges. Two ER networks are randomly connected by 10 edges. Then after applying the 3-step strategy to improve the robustness, the robustness of network increases from 0.44869 to 0.47926 (increases by 6.81%), and the modularity keeps its value 0.49502.
According to the above experimental results, we can see that the proposed method can effectively improve the robustness when the communities have different connection density. The increase of robustness in experiment 2 is lower than that in experiment 1, in that the network of experiment 1 is so fragile that it is easier to improve the robustness.

Figures

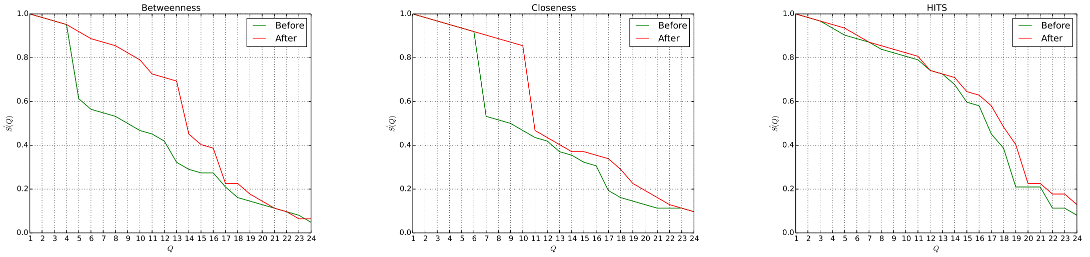

(a) (b) (c)

Figure A. Robustness against betweenness, closeness and HITS attack on the dolphin network and its corresponding improved network. Betweenness is the most harmful attack strategy. After only 5 attacks, the network breaks into pieces. In contrast to the betweenness attack strategy, the network can withstand 7 closeness attacks before it breaks into pieces. Closeness is a more harmful attack strategy than HITS. The reason is that HITS compiles two ranking vectors: hubs and authorities, however, on undirected networks, the hubs and authorities gain the same score, while the scores for hubs and authorities are different when the network is directed.

Tables

Table A. Properties of Original and Improved Networks

Network	Vertices	Edges	Category	Clustering coefficient	Diameter	Radius	Characteristic path length	Modularity	Robustness
Airline	332	2126	original	0.625	6	3	2.738	0.362	0.107
			improved	0.487	7	4	2.726	0.292	0.159
Politics books	105	441	original	0.488	7	4	3.079	0.519	0.248
			improved	0.341	5	3	2.597	0.528	0.301
Karate	34	78	original	0.571	5	3	2.408	0.415	0.126
			improved	0.427	4	3	2.267	0.34	0.177
Dolphin	62	159	original	0.259	8	5	3.357	0.527	0.22
			improved	0.219	6	4	3.002	0.528	0.373
Football	105	615	original	0.403	4	3	2.58	0.604	0.419
			improved	0.313	4	3	2.406	0.546	0.438

References A

1. Maslov S, Sneppen K (2002) Specificity and stability in topology of protein networks. *Science* 296:910–913.

2. Schneider CM, Moreira AA, Andrade JS, Havlin S, Herrmann HJ (2011) Mitigation of malicious attacks on networks. *Proceedings of the National Academy of Sciences* 108:3838–3841.
